# Paradigm Shift in Treatment of Alzheimer's Disease: Zinc Therapy Now a Conscientious Choice for Care of Individual Patients

**DOI:** 10.4061/2011/492686

**Published:** 2011-09-22

**Authors:** Tjaard U. Hoogenraad

**Affiliations:** Department of Neurology, University Medical Centre, Utrecht, 3941 VD 20 Utrecht, The Netherlands

## Abstract

Breakthrough in treatment of Alzheimer's disease with a shift from irrational dangerous chelation therapy to rational safe evidence based oral zinc therapy. Evidence based medicine: After synthesizing the best available clinical evidence I conclude that oral zinc therapy is a conscientious choice for treatment of free copper toxicosis in individual patients with Alzheimer's disease. Hypothesis 1: Age related free copper toxicosis is a causal factor in pathogenesis of Alzheimer's disease. There are 2 neurodegenerative diseases with abnormalities in copper metabolism: (a) the juvenile form with degeneration in the basal ganglia (Wilson's disease) and (b) the age related form with cortical neurodegeneration (Alzheimer's disease). Initially the hypothesis has been that neurodegeneration was caused by accumulation of copper in the brain but later experiences with treatment of Wilson's disease led to the conviction that free plasma copper is the toxic form of copper: it catalyzes amyloid formation thereby generating oxidative stress, free radicals and degeneration of cortical neurons. Hypothesis 2: Oral zinc therapy is an effective and safe treatment of free copper toxicosis in Alzheimer's disease. Proposed dosage: 50 mg elementary zinc/day. Warning: Chelation therapy is irrational and dangerous in treatment of copper toxicosis in Alzheimer's disease.

## 1. Introduction

**Title of the Study**

The title: “Paradigm shift in treatment of Alzheimer's disease: zinc therapy a conscientious choice for care in individual patients” is comparable with the title of the article titled: “Paradigm shift in treatment of Wilson's disease: zinc therapy now treatment of choice” that I published in Brain and Development in 2006 [1]. The title preludes on the idea that lessons learnt from earlier clinical studies on zinc therapy in patients with free copper toxicosis in Wilson's disease have been very helpful in making the conscientious choice for zinc therapy for individual patients with Alzheimer's disease now.

**Aim of the Current Review**

The purpose of this analytic review is threefold: (1) to cumulate evidence for the hypothesis that Alzheimer's disease caused by an age-related disturbance of copper metabolism (type 2 free copper toxicosis) that leads to oxidative stress and neurodegeneration. (2) to provide evidence for the hypothesis that causal treatment of individual patients with Alzheimer's disease can best be done with antioxidative oral zinc therapy. (3) to warn for the tragic misconception that chelating agents are qualified for treatment of free copper toxicosis.

**Alzheimer's Disease and Copper Metabolism**

This disorder is a common neurological disease and affects about 250,000 individuals in the Netherlands. Neuropathological research has shown that the plaques detected in the brain of patients with Alzheimer's disease contain deposits of amyloid and abnormal neurofibrils, and the cortical neurons to be withered. The prognosis of Alzheimer's disease is poor. The disease is slowly progressive and spontaneous recovery has never been documented. It is essential to clarify the pathogenesis of the disease, to enable early diagnosis of the disease by means of laboratory testing [2]. As long as the cause of the disease is not exactly known, causal therapy is not possible.

Inflammatory, vascular, and genetic factors are probably important to the disease pathogenesis, but metals, especially “free-metals” like free copper and free iron may be involved in catalyzing amyloidosis and catalyzing the Fenton's reaction, leading to the generation of free radicals like hydrogen peroxide (H_2_O_2_) that can damage neurons. Specifically, free copper is an exchangeable pool of copper in serum which is probably in a Cu(I) oxidative state and is loosely bound and exchanged among amino acids, small peptides, albumin, and alpha 2 macroglobulin. This type of copper is intrinsically toxic since it can enter Fenton-like reactions triggering free radical generation [3]. Moreover, for its low-molecular-weight nature it can easily cross the blood-brain barrier as previously [4] and very recently [5] demonstrated in diverse experimental models *in vivo*. In Wilson's disease degeneration upon free copper excess primary hits the liver, the organ which tightly controls metal homeostasis in the body. This is true in cases of severe impairment of the ATPase 7B function, which have usually a juvenile presentation [3]. Free copper toxicosis in AD is sensitively milder, even though very mild effects on AD liver have been reported [6, 7].

A study has shown that the levels of the metal-binding protein metallothionein may be reduced in Alzheimer's disease [8], and another neurodegenerative disease, hepatolenticular degeneration or Wilson's disease is known to be caused by free copper poisoning [9, 10].

While a debate did exist for many years concerning a positive or negative effect of plasma copper on AD, free copper results from clinical studies carried out on Alzheimer's disease patients so far are univocal, demonstrating a detrimental effect of this type of copper on Alzheimer's disease worsening. The works from Squitti and colleagues have shown elevated levels of free copper specifically in Alzheimer's disease, that the free copper disarrangement correlated with worsen clinical status of Alzheimer's disease patients, with a worsen prognosis, and very recently with a status of mild cognitive impairment [11–16]. These studies have been confirmed by other groups from diverse countries [17, 18]. 

Danzeisen and colleagues [19] reported that free copper is not a suitable marker for copper, apparently questioning results from other authors [6, 13], although no authors ever took into account this possibility. Free copper is, instead, one of the seven diagnostic tests which may help to ensure that the correct diagnosis of Wilson's disease is made [3, 9]. On the other hand, copper concentration in the liver is the most robust marker of copper status in the body, but the measurement is invasive. Ceruloplasmin can be a quite good marker of body copper status, especially in condition of a copper deficiency, as for example on a copper-deficient regimen, which results in ceruloplasmin serum decreases [20].

## 2. Neurodegeneration and Copper Metabolism

There are many neurodegenerative disorders, such as Alzheimer's disease, lenticular degeneration in Wilson's disease, Parkinson's disease, Huntington's disease, amyotrophic lateral sclerosis, age-related macular degeneration, and hereditary ataxia. The pathogenesis of most of these diseases is unknown. Wilson's disease is the only exception: neurodegeneration of the basal ganglia of the brain is caused by free copper toxicosis [3, 10]. Wilson's disease became the first neurodegenerative disease for which causal treatment was discovered. Causal therapy is possible: zinc therapy is a safe and effective treatment of free copper toxicosis [21].

## 3. Amyloidosis and Free Copper

The characteristic Alzheimer plaques contain accumulations of amyloid. These deposits also have excess copper and iron. Research into development of drugs for Alzheimer's disease target at amyloid accumulation in the brain. Amyloid is also found in the macula of patients with age-related macular degeneration. Zinc supplementation aiming at decrease of oxidative stress has been found to be effective [22].

**Amyloidosis in Type 2 Diabetes Mellitus**

amyloid deposits accumulate in plaques in pancreatic cells, a process that is catalyzed by free copper. Hydrogen peroxide is generated during the aggregation of amylin peptide into amyloid. The formation of hydrogen peroxide is greatly stimulated by free copper and could cause progressive degeneration of islet cells in type 2 diabetes mellitus [23].

## 4. Chelation Therapy in Wilson's Disease

Initially, Wilson's disease was thought to be caused by toxic effects of copper deposits that had been accumulated in the basal ganglia, and metalchelators such as BAL and penicillamine were given in order to liberate copper from the stores and increase the excretion of copper via the urine [9]. Indeed for about 50 years penicillamine has been used for the treatment of copper accumulated in Wilson's disease, and the toxic chelating agent became accepted worldwide as the treatment of choice: survival was thought to be prolonged by penicillamine and signs of copper accumulation, such as Kayser-Fleischer rings, diminished or disappeared [9].

**The Chelation Therapy Fallacy**

Evidence-based medicine disputes efficacy, safety, and much of the theory behind chelation therapy. The fatal flaw of the copper chelating strategy is that it is based on the method of induction and that it was developed without any deductive, evidence-based reasoning and without any clinical trials having been performed.

## 5. Chelation Therapy in Alzheimer's Disease

An article on nanochelationtherapy published in September 2005 [24] first drew my attention to the disturbance of copper homeostasis in Alzheimer's disease. The authors referred to two articles published by Squitti and colleagues [10, 11]. In one of these studies, Squitti et al. [25] had measured serum copper concentrations in 47 patients with Alzheimer's disease, 24 patients with vascular dementia, and 44 healthy controls. Mean free copper concentrations in the Alzheimer's disease patients were significantly higher than in the vascular dementia patient group and control group. The authors speculated that the raised copper concentrations could play a role in the pathogenesis of the degenerative process. In an earlier study, the same authors had described the results of a double-blind, placebo-controlled pilot study of the effects of chelation therapy with penicillamine (600 mg/day) for 6 months in patients with Alzheimer's disease [25]. At that time, it was not appreciated that patients could be suffering from free copper poisoning. Instead, treatment was focused on copper accumulation, and the aim of treatment was to increase the excretion of copper in urine, to reduce laboratory measures of oxidative stress, and to slow the progression of cognitive deterioration, as measured with neuropsychological tests. Thirty-four patients took part in the study: 17 received placebo and 17 received penicillamine. Only 9 patients in both groups completed the study. Of the patients treated with penicillamine, 1 died of a heart infarct and 4 experienced serious adverse events. The pilot study was stopped prematurely by the ethics committee because there were too many adverse events. The authors concluded that penicillamine had not slowed the clinical progression of the disease and that a less harmful chelating agent should be sought. As far as I am aware, Squitti and her colleagues are the first investigators to have performed a controlled clinical study on efficacy and side effects of chelation therapy.

## 6. Evidence-Based Medicine in Alzheimer's Disease

Making decisions on causal drug therapy in individual patients with Alzheimer's disease is hampered by the fact that randomized clinical trials to base the decisions on are not easy to find. Nevertheless such trials are essential to identify dangerous and worthless treatments. I found that he clinical trial described by Squitti et al. in 2002 [25] is such a trial. It was a real eye opener, it identified the paradoxical effect of penicillamine in Alzheimer's disease: the trial had to be stopped by the ethical board because of the severe side effects of the chelating agent. This paradoxical effect of penicillamine in Alzheimer's disease reminded me of the dangerous paradoxical effect seen in patients with free copper toxicosis in Wilson's disease and it stimulated me to develop the hypothesis that free copper toxicosis might play a causal role in Alzheimer's disease.

In my opinion, chelating agents, including PBT1 (clioquinol) and PBT2 [26, 27], should be tested no more on their effect on safety in treatment of patients with Alzheimer's disease. An ethical committee involved in the preparation of such a clinical trial should not give permission for it being performed. Since we know that copper metabolism is disturbed in Alzheimer's disease and since we know that a randomized clinical trial with the chelating agent penicillamine had to be stopped by the ethical committee because of severe side effects [25], it is irresponsible to ask patients to give permission to participate in a clinical trial with the chelating agent clioquinol.

## 7. Zinc Therapy

A randomized clinical trial testing a clear hypothesis is needed before conclusions can be drawn about the value of zinc supplements in the treatment of Alzheimer's disease. Such a clinical trial could be set up on lines similar to the randomized clinical trial of penicillamine performed in 2002 [25]. The effect of a low dose of zinc (50 mg/day) on the free copper concentration in serum, the urinary excretion of copper, laboratory markers of oxidative stress, and cognitive functions could be investigated in a blind, placebo-controlled trial. In conclusion, the work of Squitti et al. has provided data to justify the hypothesis that free copper concentrations are raised in patients with Alzheimer's disease. In my opinion, it is justified, and even desirable, to test in a controlled study the hypothesis that the free copper poisoning of patients with Alzheimer's disease is amenable to zinc therapy. A previous study suggested potential benefit of a zinc therapy in AD [28]. Moreover, a very preliminary study with Zinc therapy gave positive results in counteracting AD progression [28]. Specifically, a previous study suggested potential benefit of a zinc therapy in AD [28]. Ten patients were treated, all of them receiving 50 mg of oral zinc *bis*-(dlhydrogen aspartate) three times daily obtaining improvement of memory, understanding, communication, and social contact in eight patients, as stated by the author [28]. The discontinuation of the treatment decreased and even reversed the improvement, in all patients. However, these conclusions have to be taken very cautiously. Even though these limitations, a phase II, multicentre, prospective, randomized, double-blind, placebo-controlled, parallel group study conducted with patients presenting a diagnosis of mild to moderate clinical trial with the following characteristics: (a) a treatment duration of at least one full year, as expected with curative compound use versus symptomatic approaches; (b) a patient-inclusion criterion based on individual serum Cu-dysfunction evidence; (c) monitoring of Cu bioavailability throughout the study with detection of Cu metabolism markers such as free Cu, ceruloplasmin, or Cu/Zn superoxide dismutase levels, to prevent adverse events; (d) statistical power (at least 75 patients per arm, placebo and treated); a dose of zinc sulphate 400–1200 mg/day, can be proposed.

## 8. Additional Paragraph on Very Recent Studies on Alzheimer Research

Two very recent articles [29, 30] cast biometals in the scenario of the variegated milieu of biomarkers of cerebrospinal fluid (CSF), imaging biomarkers and peripheral biomarkers of Alzheimer's disease under investigation. In recent years, concentrations of CSF amyloid-*β*42, total Tau, and phosphorylated Tau, *in vivo* molecular imaging of intracerebral beta-amyloid load (by the Pittsburgh Compound-B: PiB-PET), structural and functional neuroimaging changes, or peripheral biomarkers (including inflammatory markers interleukins, cytokines, oxidative stress compounds isoprostanes, plasma APP markers (BACE 1) and other markers of synaptic damage/neurodegeneration) [31–33] have been repetitively reported as differentiating factors of Alzheimer's disease which also reflect core pathological changes of the disease, eventually leading to full dementia. The two papers, which disclose the potentiality of metallochemistry in clinical studies on Alzheimer's disease patients [29, 30] have been preceded by numerous and heterogeneous reports exploring the reliability of some metals, particularly copper, in characterizing Alzheimer's disease patients and cognitive worsening. Besides the so many case-control studies evaluated in a recent meta-analyses by Bucossi and coworkers [29], at least two prospective studies did demonstrate the relevant influence of copper dysfunction of cognitive status. Specifically, (i) a community-based prospective study exploring cognitive functions in a cohort of 3,718 elderly individuals [34], revealed that a diet high in copper combined with a high dietary intake of saturated fats associated with a faster rate of cognitive decline, specifically with a lost cognition at a rate three-times higher than expected [34]; (ii) a study on 81 subjects with mild to moderate Alzheimer's disease patients, clinically followed up for 1 year[15]finding that higher levels of copper at the baseline correlated with a worsened cognitive status at 1 year. These results are in line with the Rancho and Bernardo study [35] which evaluated 602 men and 849 women for metals (Cu, Fe, and Zn) in association with cognitive performance and revealed that women had worsened performance in total and long-term word (but not short) retention and higher plasma copper levels, as evaluated by the Buschke-Fuld Selective Reminding Test, as well as poorer concentration abilities, as tested by the Blessed Information-Memory-Concentration Test. In their study, Bucossi and coworkers [29] analyzed in a meta-analysis design, data from all the serum, plasma, and CSF case-control studies published since 1983 on Alzheimer's disease patients, to gain an objective evaluation of whether systemic copper variation were associated with Alzheimer's disease. Data from 21 studies on serum copper and 5 studies on plasma copper were merged for a pooled total of 966 Alzheimer's disease patients and 831 controls which were enough to draw the conclusion that Alzheimer's disease patients had higher levels of serum copper than healthy controls, sufficient to unambiguously distinguish Alzheimer's disease patients from healthy controls. From a totally different perspective the same authors challenged the copper hypothesis in Alzheimer's disease, screening chromosomes from Alzheimer's disease patients for Wilson's disease mutations or polymorphisms [30]. They found that the Wilson's disease ATP7B gene—which is a tight control balance regulator for free copper levels in the body—presents susceptibility loci for late-onset Alzheimer's disease [30]. As stated by the authors, they explored Alzheimer's disease chromosomes with a hypothesis-driven candidate gene association study, to verify whether the Wilson's disease ATP7B gene had susceptibility loci for late-onset Alzheimer's disease, assuming that the free copper disproportion is true and specifically associated with AD. Moreover, the authors gained further results on an additional exon 12 single nucleotide polymorphism (SNP) of the ATP7B gene associated with Alzheimer's disease and in linkage disequilibrium with the exon 10 SNP one, suggesting that exon 12 or something very close to it can be a susceptibility locus for Alzheimer's disease (R. Squitti personal communication). It would be surprising that genome-wide association studies (GWASs) carried out so far have never found an association between Alzheimer's disease and the 13q14.3 DNA region where the ATP7B gene lies. However, to this regard, another paradigm shift recently proposed should to be considered [36]. In fact, current experience with GWASs suggests that rarer variants that are, actually, hard to detect by GWASs, may account for the missing hereditability of Alzheimer's disease, estimated around 58–79%, and which plays a role in the development and progression of Alzheimer's disease [37]. The paradigm recently proposed of a shift from the “common disease—common variant hypothesis “to a “common disease—multiple rare variants hypothesis” may suitably fit to the ATP7B gene association with Alzheimer's disease, since the gene is highly polymorphic but at the same time harbors rare mutations, that in homozygous or heterozygous compounds trait are causative of Wilson's disease (Squitti R. personal communication). In other words, it seems not premature to sustain the hypothesis that type 2 free copper toxicosis may play a causal role in age related Alzheimer's dementia.

**Glossary**

**Paradigm Shift**

a radical change in thinking from an accepted point of view to a new one. For instance, as to neurodegeneration: from no causal treatment available to zinc therapy for treatment of free copper toxicosis.

**Deductive Method**

This evidence based method aims at identifying errors and fallacies. Randomized clinical trials are based on the deductive method.

**Evidence Based Medicine**

The conscientious use of current best deductive evidence in making decisions about the care of individual patients.

**Free Copper**

The small portion of plasma copper that is not bound to ceruloplasmin. Free plasma copper is the toxic form of the metal. Copper accumulated in deposits in Alzheimer plaques is not toxic.

**Inductive Method**

This method aims at verification of the theory but not at detecting its errors. It may lead to entrapment in fallacies.

**Penicillamine Fallacy**

The tragic, inductive method based misconception that treatment of copper toxicosis can best be started with a chelating agent like penicillamine.

**Seductive Method**

The unscientific method of choosing a therapy simply on the basis of expert opinion, pharmaceutical representatives or advertisements.

**Type (Juvenile) Free Copper Toxicosis in Wilson's Disease**

The type of free copper toxicosis causing oxidative stress with free radicals leading to neurodegeneration especially in the basal ganglia.

**Type (Age Related, Senile) Free Copper Toxicosis in Alzheimer Disease**

Conscientious analytic cumulating of the available evidence has led to the hypothesis that free copper toxicosis does catalyze formation of amyloid in plaques and oxidative stress causing neurodegeneration in Alzheimer's disease.

**Zinc Therapy**

A randomized clinical trial testing a clear hypothesis is needed before conclusions can be drawn about the value of zinc supplements in the treatment of Alzheimer's disease. Such a clinical trial could be set up on lines similar to the randomized clinical trial of penicillamine performed in 2002 [25]. The effect of a low dose of zinc (50 mg/day) on the free copper concentration in serum, the urinary excretion of copper, laboratory markers of oxidative stress, and cognitive functions could be investigated in a blind, placebo-controlled trial. In conclusion, the work of Squitti et al. has provided data to justify the hypothesis that free copper concentrations are raised in patients with Alzheimer's disease. In my opinion, it is justified, and even desirable, to test in a controlled study the hypothesis that the free copper poisoning of patients with Alzheimer's disease is amenable to zinc therapy. A previous study suggested potential benefit of a zinc therapy in AD [28]. Moreover, a very preliminary study with Zinc therapy gave positive results in counteracting AD progression [28]. Specifically, a previous study suggested potential benefit of a zinc therapy in AD [28]. Ten patients were treated, all of them receiving 50 mg of oral Zinc bis-(DLhydrogenaspartate) three times daily obtaining improvement of memory, understanding, communication, and social contact in eight patients, as stated by the author [28]. The discontinuation of the treatment decreased and even reversed the improvement, in all patients. However, these conclusions have to be taken very cautiously. Even though these limitations, a phase II, multicentre, prospective, randomized, double-blind, placebo-controlled, parallel group study conducted with patients presenting a diagnosis of mild to moderate clinical trial with the following characteristics: (a) a treatment duration of at least one full year, as expected with curative compound use versus symptomatic approaches; (b) a patient-inclusion criterion based on individual serum Cu-dysfunction evidence; (c) monitoring of Cu bioavailability throughout the study with detection of Cu metabolism markers such as free Cu, ceruloplasmin, or Cu/Zn superoxide dismutase levels, to prevent adverse events; (d) statistical power (at least 75 patients per arm, placebo and treated); a dose of Zinc Sulphate 400–1200 mg/day, can be proposed.
